# Performance Evaluation of Novaplex^TM^ Multiplex Real-Time PCR Assay for Detection of *Streptococcus agalactiae* Serotypes

**DOI:** 10.3390/microorganisms12102043

**Published:** 2024-10-10

**Authors:** Mallikarjun Handigund, Jaehyeon Lee

**Affiliations:** 1 Department of Laboratory Medicine, Jeonbuk National University Medical School and Hospital, Jeonju 54907, Republic of Korea; ecoarjun156@jbnu.ac.kr; 2 Research Institute of Clinical Medicine of Jeonbuk National University, Biomedical Research Institute of Jeonbuk National University Hospital, Jeonju 54907, Republic of Korea

**Keywords:** group B streptococcus, multiplex PCR, *Streptococcus agalactiae*, serotype, NovaPCR

## Abstract

*Streptococcus agalactiae*, or group B streptococcus (GBS), is a Gram-positive pathogen with an extended track record of colonization in the gastrointestinal and genitourinary tracts. GBS can induce disease in individuals across all age demographics, yet it predominantly triggers infections in neonates and the elderly. Identification of the serotype is vital for effective management of the disease as it provides critical information for clinicians on the cause of the disease. In this study, we evaluated the rapid, simple, and easy-to-adopt multiplex real-time PCR technique, Novaplex^TM^ (NovaPCR). A total of 131 clinical isolates of different serotypes were tested using NovaPCR. Observations revealed that 129 isolates showed the same observations as LA and conventional mPCR. NovaPCR accurately identified serotypes IV and V, which were first classified as serotype Ia in the LA test and mPCR, and the difference between the traditional (LA test and mPCR) and NovaPCR methods is only 1.52%. Accurate serotype identification is helpful for monitoring the epidemics and achieving optimal clinical outcomes, and NovaPCR showed a reliable, fast, easy-to-interpret, and cost-efficient performance in GBS serotyping.

## 1. Introduction

*Streptococcus agalactiae*, or group B streptococcus (GBS), is a long-familiar Gram-positive pathogen that frequently colonizes gastrointestinal and genitourinary tracts [1]. GBS can cause illness in individuals of all age groups but primarily causes infections in neonates and the elderly [2,3]. Moreover, it is an emerging infectious disease in adults with pre-existing medical conditions or immunocompromised conditions [4,5]. Hence, GBS is now considered an important pathogen among immunosuppressed individuals. Thus, it is crucial to perform screening tests to identify group B streptococcus (GBS) in pregnant women, as it poses a significant threat to both newborns and the elderly. GBS virulence ability is believed to be associated with its capsular polysaccharide. The genes in the *cps* locus determine the serotype of a GBS. Until now, 10 serotype variants that differ in their virulence patterns have been recognized. The serotypes Ia, Ib, II, III, IV, V, VI, VII, VIII, and IX are identified depending on the capsular polysaccharide [6]. Generally, these serotypes demonstrate varying prevalence, geographic distribution, and virulence. For instance, serotype III is linked to neonatal invasive diseases [7,8]. In contrast, serotype V is linked to various infections, particularly in older patients with skin and soft tissue infections [9,10]. Thus, detection and understanding of the distribution of serotypes may assist clinicians in identifying the risk factors and help them to treat patients accordingly. Furthermore, monitoring the serotype is crucial for vaccine development and clinical epidemic research. For example, immunization with capsular polysaccharide (CPS)-protein conjugates is one of the possible solutions to decrease the GBS infection, and GBS conjugate vaccines covering the majority of serotypes are at the developmental stage [11]. Notably, it is widely recognized that serotypes can vary in their virulence characteristics, affecting the degree of the infection and the clinical outcomes [12,13,14]. Therefore, the identification of serotypes becomes clinically significant for clinicians for potential treatment.

Traditionally, the Lancefield precipitation test (LP test) has long been regarded as the standard method for GBS serotyping. In this method, specific antigens from the surface of GBS are extracted and allowed to react with serotype-specific antisera to detect the serotype. However, the extraction of specific antigens is a laborious process, which makes it impractical to analyze a large number of samples [15]. Also, the LP test can yield false-negative or false-positive results due to cross-reactivity or weak antigen-antibody interactions [16]. Additionally, the LP test has been rendered obsolete with the development of the latex agglutination test (LA test), flow cytometry [17], and PCR-based assay [3,18]. The flow cytometric technique uses fluorescent antibodies to identify the GBS serotypes. This method offers high sensitivity, specificity, multiplexing, and quantitative analysis. However, it requires technical expertise and involves complex sample preparation procedures. In contrast, the LA test is a rapid and less expensive technique, but it is laborious when analyzing a large number of samples. Thus, the high cost of consumables and equipment and the need for specialized technicians make both methods less applicable for large-scale testing. Therefore, rapid, economical, and precision-oriented methods are essential for the identification of GBS serotypes. Further, PCR-based techniques look promising, as they offer rapid results and adaptability for detecting multiple or emerging serotypes, as well as the simultaneous analysis of multiple samples, which enables short turnaround times. Still, several PCR assays demonstrated inconsistent results with the LA test, complicating the interpretation. Multiplex PCR, a technique that allows the simultaneous amplification of multiple target DNA sequences, is the preferred method by the Centers for Disease Control and Prevention (CDC) [19]. Precise design and optimization of primers are crucial for the performance of traditional multiplex PCR methods in detecting serotypes, but these steps also add variability. Additionally, the interpretation of multiplex PCR results is complex. In contrast, multiplex PCR kits deliver standardized and readily available reagents for the simultaneous detection of multiple targets, assuring consistent outcomes and user-friendly application. Thus, evaluation of newly developed rapid multiplex PCR kits is crucial for ensuring their accuracy, reliability, and clinical applicability. However, to our knowledge, there were no commercially available assays for simultaneous detection of the GBS serotypes. Even if such assays are available, they were intended for research purpose only. Herein, we evaluated the rapid, simple, and easy-to-adopt multiplex real-time PCR technique called Novaplex^TM^ (NovaPCR, Seegene Inc., Seoul, Republic of Korea) for clinically relevant serotypes.

## 2. Materials and Methods

### 2.1. Sample Collection

A total of 131 GBS isolates collected from clinical samples in the Department of Laboratory Medicine, JBNU hospital, between 2008 and 2018 were enrolled in this study to identify GBS serotypes using NovaPCR. These 131 samples were collected from various locations: urine (33), blood (26), spot urine (15), vaginal discharge (13), pus (12), tissues (6), closed pus (4), open pus (3), cerebrospinal fluid (2), NCT urine (2), urine catheter (2), eye discharge (1), sputum (1), and others (7). The collected samples were identified with automated culture systems Vitek 2 System (BioMérieux Inc., Hazelwood, MO, USA) and VitekMS (BioMérieux Inc., Marcy L’Étoile, France), and all the isolates were stored in skim milk at −80 °C. Subsequently, collected samples were cultured in blood agar plates (BAP) and incubated at 35 °C for 16–24 h before experiments. The bacterial strains used in this investigation were collected after clinical practice for diagnosis or for surveillance. Additionally, the samples used for the investigation are entirely de-identified, ensuring the non-traceability of their origin. All bacterial isolate collection and storage procedures were performed in accordance with the Micro Bank at Jeonbuk National University Hospital (JBUH) and the department of laboratory medicine in JBUH.

### 2.2. Serotyping bt Latex Agglutination (LA)

The *S. agalactiae* strains were cultured on BAP. All the selected GBS isolates were serotyped by LA test (ImmuLex^TM^ Streptococcus-B kit, SSI Diagnostica, Hillerød, Denmark) according to the manufacturer’s instructions. Briefly, 10 µL of RNase-free water and a single bacterial colony were applied to a reaction card provided by manufacturers and mixed thoroughly with a drop (2 µL) of latex suspension. Then, the reaction card was rotated slowly and observed for agglutination. A positive reaction was noted when distinct and visible agglutination occurred within a time frame of 30 s.

### 2.3. Extraction of Nucleic Acid and Multiplex Polymerase Chain Reaction (mPCR)

Total DNA was extracted by the modified boiling method [20]. Briefly, two to three colonies from BAP were transferred to an Eppendorf tube containing 1 mL of distilled water. Then, samples were mixed thoroughly by vertexing and centrifuged at 13,000 rpm for 10 min to collect the pellet. Then, 100 µL of DNA extraction buffer (Seegene Inc., Seoul, Republic of Korea) was added, and the suspension was boiled at 95 °C for 20 min and centrifuged for 10 min. The supernatant was used as the template for PCR to detect the GBS serotype. Extracted DNA was stored at −80 °C or used immediately.

We performed the conventional multiplex PCR (mPCR) with extracted DNA using the same set of primers as described previously [21]. Briefly, samples were amplified by an initial denaturation step at 95 °C for 5 min. Then, the PCR protocol; 15 cycles of 95 °C for 60 s, 54 °C for 60 s, and 72 °C for 2 min, and following this, an additional 25 cycles of 95 °C for 60 s, 56 °C for 60 s, and 72 °C for 2 min were used. The final extension was performed at 72 °C for 10 min. All the products were evaluated using 1.5% agarose gel. All the primer sequences used in the study are listed in Table 1.

### 2.4. Multiplex Real-Time PCR

Nucleic acids were extracted from using 330 µL of diluted samples from two to three colonies using Microlab NIMBUS IVD platform (Seegene Inc., Seoul, Republic of Korea) with STARMag 96X4 Universal Cartridge kit (Seegene Inc., Seoul, Republic of Korea). All the extraction procedures were carried out in accordance with the manufacturer’s instructions. The final elution volume was 100 µL. The multiplex real-time PCR was done with a Novaplex^TM^ multiplex real-time PCR kit (NovaPCR, Seegene) according to the manufacturer’s instructions. The samples were amplified with a step for 15 min at 95 °C, followed by 45 cycles of 95 °C for 10 s, 60 °C for 15 s, and 72 °C for 10 s. Fluorescence was detected at 60 °C and 72 °C. All real-time PCR runs were performed on CFX96**^TM^** (Bio-Rad Laboratories, Hercules, CA, USA). GBS type Ia and Ib were recognized by fluorophore FAM (Fluorescein amidite), GBS type II and VI by fluorophore HEX (Hexachlorofluorescein), GBS type III and V by fluorophore Cal Red 610, GBS type IV, VII, VIII, and IX by fluorophore Quasar 705, fluorophore Quasar 670 identified internal control (IC), and common GBS region. All the samples were done on 8-strip tubes and 96-well plates. All the data were analyzed by Seegene Viewer (Seegene Inc., Seoul, Republic of Korea), an automated data analysis application for multiplex real-time PCR. Primers/probes for Novaplex^TM^ multiplex PCR reactions are withheld, as this method is deemed a commercial product.

### 2.5. Sensitivity and Specificity of NovaPCR

In this study, 1000-fold diluted DNA was used to evaluate the sensitivity of the NovaPCR. Generally, DNA may exist in various concentrations and can degrade during storage. Thus, testing the NovaPCR performance with such diluted DNA indicates its robustness across different sample concentrations. Here, the study used LA and conventional multiplex PCR as reference methods to confirm the efficiency of NovaPCR. Also, for the analytical specificity, *Bacillus cereus*, *Enterobacter cloacae*, *Enterobacter hormaechei*, *E coli*, *Lactobacillus salivarius*, *Lactococcus garvieae*, *Staphylococcus epidermidis*, *Staphylococcus gordonii*, and *Streptococcus pneumoniae* isolates were tested, and those strains were received from Jeonbuk National University Hospital Culture collection for pathogens to verify the potential interferences by non-target species. This step was performed with the same primer and probe set under the conditions mentioned in NovaPCR with DNA from non-target clinical isolates.

### 2.6. Interpretation and Statistical Analysis

The observations acquired with mPCR were compared to those obtained from the LA test to validate the accuracy of the serotype identification. A comparison between the two methods assisted in obtaining consistent, accurate, and reliable serotype identification. A true serotype was considered when the isolates showed positive for a specific serotype in both methods. Next, we analyzed the sensitivity, specificity, and agreement of NovaPCR based on the true serotypes. When there were discrepancies between the mPCR, latex agglutination test, and NovaPCR, the strains were sequenced, and the serotypes were confirmed based on the sequence results. All statistical analyses were conducted with MedCalc Statistical Software ver.19.2.1. (MedCalc Software Ltd., Ostend, Belgium).

## 3. Results

### 3.1. Serotype Distribution by LA Test

**Figure 1:**
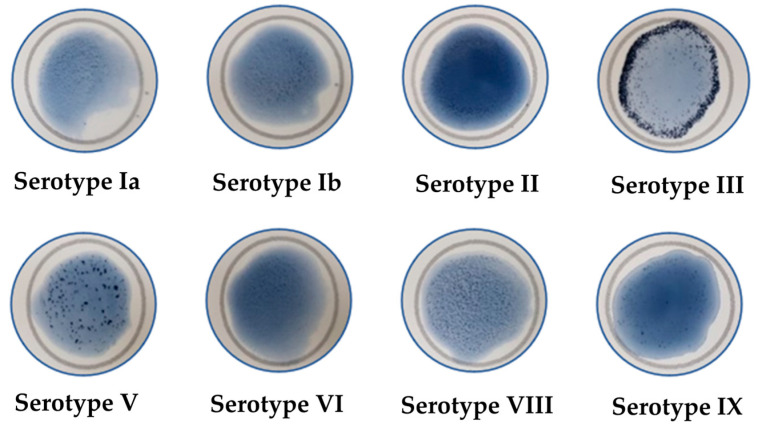
Representative images showing positive LA tests for specific serotypes.

The LA test is considered an efficient and standardized technique used for serotyping GBS. It has been demonstrated to be serotype-specific and can effectively detect most GBS strains. In this study, the LA test identified 8 serotypes. The distribution of the serotypes was as follows: Ia, 15.26% (20/131); Ib, 15.26% (20/131); II, 7.63% (10/131); III, 15.26% (20/131); V, 16.03% (21/131); VI, 15.26% (20/131); VIII, 15.26% (20/131); IX, 0.76% (1/131). However, we could not acquire serotypes IV and VII through the LA test because their incidences are extremely low. Figure 1 demonstrates a visible clumping (agglutination) in the respective serotype within 30 sec of beginning the card rotation. Specifically, serotypes III, V, and VIII exhibited strong agglutination, while others demonstrated moderate agglutination. All the images were captured within the manufacturer’s interpretation time frame. All the identified serotypes from different samples are listed in Table 2.

The Table 2 observations demonstrate the distribution of various serotypes across different specimen types, with Serotypes V and VI being the most frequent (21 and 20 occurrences, respectively). At the same time, serotypes IV and VII are absent. Serotype V is found across all specimens, mainly in urine samples, while Serotype IX is the least frequent, with just 1 occurrence in urine. Serotypes Ia, Ib, and V are widely distributed, but their distribution is not even among samples. These observations suggest uneven distribution across multiple specimens.

### 3.2. Serotype Distribution by Conventional mPCR

mPCR was performed to validate its efficacy against the observations of the LA test. Here, we identified the same number of serotypes as identified in the LA test and observed 100% concordance between the mPCR and LA methods. The strong agreement between the two methods suggests their comparable reliability for serotype identification. Furthermore, these observations indicate that any of these approaches can be used based on the clinical needs. The perfect concordance also implies that there were no discrepancies or false results in either test, highlighting the accuracy and reliability of these techniques used by most diagnostic laboratories to detect serotypes.

### 3.3. Serotype Identification by NovaPCR

NovaPCR detected 9 serotypes. The distribution of the serotypes was as follows; Ia, 13.74% (18/131); Ib, 15.26% (20/131); II, 7.63% (10/131); III, 15.26% (20/131); IV, 0.76% (1/131); V, 16.79% (22/131); VI, 15.26% (20/131); VIII, 15.26% (20/131); IX, 0.76% (1/131). Interestingly, serotype IV was detected in one isolate from the urine sample, which was serotype Ia in both LA and mPCR. Thus, the total number of serotypes observed in the NovaPCR screening is 9. Furthermore, one isolate from the pus samples screened by NovaPCR revealed serotype V, whereas the LA test and mPCR identified the same sample as serotype Ia. Statistically, NovaPCR observations agree with the LA and mPCR tests, with a PPA (positive percent agreement) value of 1 and a kappa value of 1 in most samples. However, serotype Ia is the exceptional serotype, with a PPA of 0.94 and a kappa value of 0.98. Comparative observations of serotypes identified using different methods are presented in Table 3. In addition, positive percent agreement (PPA) values are shown in Table 3 to substantiate the efficacy of NovaPCR.

NovaPCR observation showed two discrepancies out of 131 samples compared to LA or mPCR serotyping. Specifically, the two samples were detected as serotype Ia in LA and mPCR tests. However, NovaPCR detected those samples as serotypes IV and V. To validate the NovaPCR observations, sequencing was performed. The sequencing results confirm those samples as serotypes IV and V, clearly indicating the efficiency of NovaPCR.

### 3.4. Sensitivity and Specificity of NovaPCR

To assess the sensitivity of the NovaPCR method, samples were serially diluted up to 1000-fold, and all dilutions across all serotypes and samples showed positive results. NovaPCR clearly detected serotypes in all the diluted samples, indicating its efficacy at lower concentrations. In a specificity test using bacteria other than GBS to verify the interference by non-target strains, NovaPCR results were observed to be negative for all the non-target strains.

## 4. Discussion

GBS is considered an asymptotic colonizer in healthy adults but can cause severe invasive infections in pregnant women, neonates, and immunosuppressed adults. The occurrence of GBS disease has seen a rise in incidence among nonpregnant adults, increasing from 3.6 to 7.3 cases per 100,000 people between 1990 and 2007 in the United States [22]. Interestingly, most affected adults have comorbidities such as heart disease, neurological disorders, diabetes, and immunocompromising conditions [23]. It is estimated to have a 2–10% fatality rate in premature neonates [24]. Also, GBS causes significant maternal morbidity and is responsible for severe infections in elderly individuals, diabetic patients, and immunocompromised individuals. The World Health Organization (WHO)-funded systematic review and meta-analysis revealed that, globally, 48.9% of invasive GBS disease was due to serotype III in neonates, followed by serotype Ia (22.9%), Ib (7%), II (6.2%), and V 9.1% [25]. Moreover, several reports suggest a correlation between different serotypes and diseases caused [26,27,28]. For instance, serotype III is frequently associated with invasive illnesses in newborns, such as sepsis and meningitis [29,30]. Furthermore, serotypes Ia, Ib, II, III, and V have been implicated with severe invasive illness. Interestingly, previous reports suggest that serotypes V, III, and Ia collectively constitute 67% of the prevalent serotypes responsible for invasive soft tissue infections [10]. Each serotype, with its unique characteristics, seemed to play a crucial role in determining and exhibiting distinct characteristics in its virulence, prevalence, and the populations it affects. The reliability and accuracy of detecting serotypes are essential for developing effective vaccines, guiding antibiotic prophylaxis, and understanding the association of serotypes with transmission patterns of specific pathogens. Accurate serotype detection becomes particularly crucial when considering epidemics. Thus, the accuracy of detection is crucial in understanding the pathogenesis and prevention of GBS infections. In this study, efforts were made to validate the novel mPCR called NovaPCR for rapid and reliable detection of GBS serotypes.

Despite advances in molecular techniques, and in the era of whole-genome sequencing [31], the diagnosis and serotyping of GBS continue to rely heavily on traditional methods such as LA and mPCR, which can be time-consuming and labor-intensive [32,33]. Although LA is widely used and considered the standard method for serotyping, it has significant limitations. The major limitations include cross-reactivity between serotypes, subjective interpretation, and the inability to type certain isolates, resulting in a substantial proportion of non-typeable strains [33,34]. Slotved et al. reported that 12.5% of isolates could not be serotyped using LA [35]. Also, LA is unsuitable for processing large volumes, it cannot be used directly with clinical samples, and its reagents are expensive, adding to the burden of detection [15]. Multiplex PCR offers improved accuracy but requires specialized equipment, expertise, and complex workflows, making it less accessible for routine clinical use. While mPCR has been developed to address some limitations of LA, it still involves labor-intensive tasks such as gel loading and aliquoting of multiple tubes, which can lead to human error and reduced cost-efficiency [36,37,38,39]. The tedious nature of these methods can delay appropriate treatment and epidemiological surveillance [40]. Breeding et al. reported that serotyping by LA can take up to 24 h, while PCR-based methods, though faster, still require several hours for results. Furthermore, discrepancies between phenotypic and genotypic methods complicate interpretation. Imperi et al. found only 71.1% agreement between LA- and PCR-based serotyping [41]. Some GBS strains show weak reactions in LA assays, leading to incorrect identification despite mPCR confirmation, possibly due to cross-reactivity or weak antigen expression among serotypes. Consequently, the analysis of LA test results may require substantial knowledge of potential cross-reactions. These challenges underscore the growing demand for streamlined, high-throughput, and automated methods to improve the speed and accuracy of GBS detection and serotyping in clinical diagnostics, particularly when rapid diagnosis is crucial for neonatal and maternal health. Real-time multiplex PCR emerges as a promising alternative, offering a straightforward, rapid, and reliable strategy to detect GBS serotypes in an easy, simple, and effective manner.

In this study, a total of 131 clinical isolates of different serotypes were tested using NovaPCR. Observations revealed that 129 isolates showed the same observations as LA and conventional mPCR. However, the two isolates in NovaPCR are distinctively different from LA and conventional mPCR. Specifically, two isolates in serotype Ia were detected as serotype IV and V in NovaPCR. To solve the discrepancies, sequencing was performed, and the results of two isolates revealed the same serotype by sequencing as observed in NovaPCR. These observations suggest that there could be potential ambiguity issues with the LA test and conventional mPCR. The serotype Ia is one of the most common and widely distributed GBS serotypes. It is frequently associated with neonatal GBS early-onset disease (EOD), which occurs within the first week of life and often leads to severe conditions such as sepsis, pneumonia, and meningitis [42,43,44]. The difference between the traditional (LA test and mPCR) and NovaPCR methods is only 1.52%, but clinically, we cannot define or measure its clinical impact on treatment and its outcome. Thus, further clinical studies are necessary to assess the clinical impact of NovaPCR’s improved efficacy in detecting GBS serotypes.

In NovaPCR, specific probes are labeled with different fluorescence dyes: FAM, HEX, Quasar 705, and Quasar 670, and employ TOCE technology. This technology streamlines the PCR process for serotyping and effectively addresses the limitations associated with target-based probe real-time PCR methods. Furthermore, all the DNA extraction and PCR preparation steps were fully automated using the Microlab NIMBUS IVD platform, ensuring fast and reliable PCR preparation. The use of advanced knowledge-based technology in NovaPCR enhances the accuracy and reliability of GBS serotype detection. In this study, NovaPCR demonstrated higher accuracy in identifying serotypes compared to LA, particularly in distinguishing serotype IV and serotype V from serotype Ia. Additionally, NovaPCR includes a dedicated viewer that allows for easy and precise interpretation of the results. These unique features make NovaPCR a dependable tool for serotyping, minimizing intra- and inter-observer variability in data interpretation. Moreover, the introduction of NovaPCR has contributed to reducing assay costs.

The NovaPCR can be helpful for detecting GBS in newborns, particularly those at risk of early-onset disease (EOD) or late-onset disease (LOD), though we did not perform the tests with clinical samples. EOD occurs within the first week of life, often within the first 24 h, and can lead to severe conditions such as sepsis, pneumonia, and meningitis. LOD typically occurs between one week and three months of age. It also presents serious health risks, including meningitis and long-term neurological complications. In this context, the ability of NovaPCR to provide rapid and accurate detection of GBS is especially critical. During labor, time is of the essence; the prompt identification of GBS colonization in the mother or the presence of GBS in the newborn can significantly influence the timing and type of interventions administered. For instance, if GBS is detected in the mother during labor, appropriate antibiotic prophylaxis can be initiated to prevent vertical transmission of the bacteria to the newborn during delivery. This timely intervention is crucial for reducing the risk of EOD. Furthermore, in cases where a newborn is suspected of having LOD, rapid GBS detection allows for swift initiation of targeted antibiotic therapy, which can be lifesaving. Delayed or inaccurate diagnosis could result in inadequate treatment, increasing the risk of severe outcomes or complications. Thus, the NovaPCR’s ability to deliver precise and fast results is essential for optimizing treatment strategies and improving clinical outcomes for both mothers and newborns.

Understanding the relationships between GBS serotypes, virulence genes, and antimicrobial resistance is critical for elucidating disease behavior and guiding treatment strategies. Specific combinations of serotypes and antimicrobial resistance patterns may enhance GBS invasiveness, complicating infection management and treatment. Previous reports suggest that a specific combination of virulence factors was predominant in invasive infection-related GBS (iGBS) [45,46]. These observations strongly indicate that these combinations may be major drivers in developing severe disease outcomes. Therefore, future investigations should target the specific combination to understand the relationship between disease and serotypes. Also, these investigations should be performed using clinical samples as they represent the actual clinical condition. However, in order to achieve success in these investigations, it is crucial to identify the serotype. NovaPCR has the ability to help with the precise identification of serotypes in various clinical samples. Collectively, NovaPCR offers a rapid, high-throughput, and reliable screening system for GBS. We acknowledge that our analysis is constrained to 131 samples and is obtained from a single hospital. Also, it needs representative clinical samples for serotypes IV, VII, and IX. Although it has shown promising results in clinical isolates, more validation utilizing clinical samples, particularly vaginal discharge or pus, is necessary before direct application.

## 5. Conclusions

NovaPCR is an advanced technique to detect GBS serotypes. It employs TOCE technology, which assists in navigating the existing technical constraints of target-based probe real-time PCR technology. Additionally, it maximizes the capabilities of real-time PCR in analyzing high multiplex data by utilizing novel components for melting temperature analysis and generating distinct signals. In this study, NovaPCR accurately identified serotypes IV and V, which were first classified as serotype Ia in the LA test and mPCR. This distinction in identification is crucial for achieving optimal clinical outcomes. Together, NovaPCR showed a reliable, fast, easy-to-interpret, and cost-efficient performance in GBS serotyping.

## Figures and Tables

**Table 1:** List of primer sequences used for multiplex conventional PCR.

Primer	Sense (5′-3′)
cpsI-Ia-6-7-F	GAATTGATAACTTTTGTGGATTGCGATGA
cpsI-6-R	CAATTCTGTCGGACTATCCTGATG
cpsI-7-R	TGTCGCTTCCACACTGAGTGTTGA
cpsL-F	CAATCCTAAGTATTTTCGGTTCATT
cpsL-R	TAGGAACATGTTCATTAACATAGC
cpsG-F	ACATGAACAGCAGTTCAACGGT
cpsG-R	ATGCTCTCCAAACTGTTCTTGT
cpsG-2-3-6-R	TCCATCTACATCTTCAATCCAAGC
cpsN-5-F	ATGCAACCAAGTGATTATCATGTA
cpsN-5-R	CTCTTCACTCTTTAGTGTAGGTAT
cpsJ-8-F	TATTTGGGAGGTAATCAAGAGACA
cpsJ-8-R	GTTTGGAGCATTCAAGATAACTCT
cpsJ-2-4-F	CATTTATTGATTCAGACGATTACATTGA
cpsJ-2-R	CCTCTTTCTCTAAAATATTCCAACC
cpsJ-4-R	CCTCAGGATATTTACGAATTCTGTA
cpsI-7-9-F	CTGTAATTGGAGGAATGTGGATCG
cpsI-9-R	AATCATCTTCATAATTTATCTCCCATT
cpsJ-Ib-F	GCAATTCTTAACAGAATATTCAGTTG
cpsJ-Ib-R	GCGTTTCTTTATCACATACTCTTG

**Table 2:** The latex agglutination test (LA test) observations on group B Streptococcus serotypes present in isolates.

Serotype/Specimen	Blood	Urine	Vaginal Discharge	Pus	Others	Total
Ia	2	4	4	4	6	20
Ib	1	7	1	4	7	20
II	2	4	2	0	2	10
III	7	7	3	0	3	20
IV	0	0	0	0	0	0
V	4	7	2	2	6	21
VI	1	9	2	5	3	20
VII	0	0	0	0	0	0
VIII	8	6	3	3	0	20
IX	0	1	0	0	0	1

**Table 3:** Comparative observations of serotypes identified using different methods along with positive percentage agreement (PPA) and kappa value.

Serotype	LA Test	mPCR	NovaPCR	Kappa Value	Agreement	
				Observed Kappa (95% CI)	Positive (%) (95% CI)	Negative (%) (95% CI)
Ia	20	20	18	0.983	94	99
Ib	20	20	20	1	100	100
II	10	10	10	1	100	100
III	20	20	20	1	100	100
IV	0	0	1	NA	NA	NA
V	21	21	22	1	100	100
VI	20	20	20	1	100	100
VII	0	0	0	NA	NA	NA
VIII	20	20	20	1	100	100
IX	1	1	1	1	100	100

## Data Availability

The datasets generated during and/or analyzed during the study are available from the corresponding author upon reasonable request.
